# Facial emotion recognition in autistic adult females correlates with alexithymia, not autism

**DOI:** 10.1177/1362361320932727

**Published:** 2020-07-21

**Authors:** Louise Ola, Fiona Gullon-Scott

**Affiliations:** Newcastle University, UK

**Keywords:** alexithymia, autism spectrum disorders, emotion recognition, females

## Abstract

Research on predominantly male autistic samples has indicated that impairments in facial emotion recognition typically associated with autism spectrum conditions are instead due to co-occurring alexithymia. However, whether this could be demonstrated using more realistic facial emotion recognition stimuli and applied to autistic females was unclear. In all, 83 females diagnosed with autism spectrum condition completed online self-report measures of autism spectrum condition severity and alexithymia, and afacial emotion recognition deficit that assessed their ability to identify multimodal displays of complex emotions. Higher levels of alexithymia, but not autism spectrum condition severity, were associated with less accurate facial emotion recognition. Difficulty identifying one’s own feelings and externally oriented thinking were the components of alexithymia that were specifically related to facial emotion recognition accuracy. However, alexithymia (and autism spectrum condition severity) was not associated with speed of emotion processing. The findings are primarily discussed with the theoretical view that perceiving and experiencing emotions share the same neural networks, thus being able to recognise one’s own emotions may facilitate the ability to recognise others’. This study is in line with previous similar research on autistic males and suggests impairments in facial emotion recognition in autistic females should be attributed to co-occurring alexithymia.

**Lay abstract:**

Research with autistic males has indicated that difficulties in recognising facial expressions of emotion, commonly associated with autism spectrum conditions, may instead be due to co-occurring alexithymia (a condition involving lack of emotional awareness, difficulty describing feelings and difficulty distinguishing feelings from physical bodily sensations) and not to do with autism. We wanted to explore if this would be true for autistic females, as well as to use more realistic stimuli for emotional expression. In all, 83 females diagnosed with autism spectrum condition completed self-report measures of autism spectrum condition traits and alexithymia and completed a visual test that assessed their ability to identify multimodal displays of complex emotions. Higher levels of alexithymia, but not autism spectrum condition features, were associated with less accuracy in identifying emotions. Difficulty identifying one’s own feelings and externally oriented thinking were the components of alexithymia that were specifically related to facial emotion recognition accuracy. However, alexithymia (and levels of autism spectrum condition traits) was not associated with speed of emotion processing. We discuss the findings in terms of possible underlying mechanisms and the implications for our understanding of emotion processing and recognition in autism.

## Introduction

Autism spectrum condition (ASC), prevalent in 1%–2% of the UK population (Baron-Cohen et al., 2009), is a heritable developmental condition characterised by impaired social and communication skills, repetitive stereotyped behaviour and restricted interests (American Psychiatric Association, 2013; ASC is synonymous with the term autism spectrum disorder used within the *Diagnostic and Statistical Manual of Mental Disorders* (5th ed.; *DSM*-5)). Symptoms of ASC are typically observable in early development, yet some social and behavioural difficulties may only be recognised when a child struggles to meet the social and educational demands of life in later childhood (Baio, 2014). As autistic individuals often experience problems with social interaction, much research has investigated whether they are impaired at processing faces – the most fundamental source of social information (Cook et al., 2013). Unlike neurotypical individuals, autistic infants typically show reduced attention to faces in their environment (Chawarska et al., 2013), while autistic adults have shown an absence of fusiform face area (FFA) activity during face-matching tasks, a brain region central to the detection and recognition of faces (Hoffman & Haxby, 2000; Schultz et al., 2003). Autistic children and adolescents are poorer than controls at matching unfamiliar faces (Riby et al., 2009; Wolf et al., 2008), and autistic adults similarly show impaired facial identity recognition (Greimel et al., 2014; Weigelt et al., 2012). However, contrasting studies have found that autistic individuals have typical FFA activity (Hadjikhani et al., 2004) and no deficit in recognising facial identity (Deruelle et al., 2004; Neil et al., 2016), thus face processing impairment in ASC is inconclusive.

Equally inconsistent findings have come from research investigating whether individuals with ASC are impaired at recognising emotion from faces. Although a deficit in facial emotion recognition (FER) is not explicitly part of the *DSM*-5 criteria for ASC, it has consistently been considered a diagnostic marker (Uljarevic & Hamilton, 2013). As FER is vital in social interaction and social–emotional reciprocity, both of which are part of ASC’s diagnostic criteria (American Psychiatric Association, 2013), impaired FER has reasonably been thought to underlie these autistic deficits (Schultz, 2005). Yet research investigating this has been mixed. Numerous studies have found that compared to controls, individuals with ASC are less accurate at labelling photographs of faces displaying the six basic emotions (happiness, sadness, anger, fear, surprise and disgust; Bölte & Poustka, 2003; Tanaka et al., 2012). However, others have found no basic FER deficit in ASC (Castelli, 2005; Homer & Rutherford, 2008). Similar inconsistencies have been seen in the recognition of complex emotions such as insincerity, as some have found impairments in ASC (Black et al., 2020; Golan et al., 2006; Peñuelas-Calvo et al., 2019), while others have not (Tracy et al., 2011). Research has also investigated whether autistic individuals are only impaired at recognising specific emotions. Yet, while there is some support for the view that they have a deficit in recognising negative emotions (Ashwin et al., 2006; Shanok et al., 2019), alternative studies have presented contrary findings (Adolphs et al., 2001). Furthermore, although a meta-analysis that pooled an abundance of these studies concluded that autistic individuals do have an FER deficit (Uljarevic & Hamilton, 2013), high statistical heterogeneity meant studies substantially varied both in whether they found a significant ASC deficit and in the strength of this effect, limiting the reliability of the overall finding. Finally, and although less researched, findings have also been inconclusive regarding whether individuals with ASC are slower at FER compared to controls. Some have found that ASC is associated with slower response times (RTs) for FER with autistic children (Bal et al., 2010), adolescents (Dalton et al., 2005) and a large sample of autistic adults (Sucksmith et al., 2013), while others have concluded that those with ASC are just as quick as controls at FER (Tracy et al., 2011). Therefore, although an FER deficit has been considered a core feature of ASC, research demonstrating this has produced considerably varied results.

To explain this inconsistency, several factors have been proposed. First, differences in participants’ age could account for why the presence (or absence) of an FER deficit in ASC varies across studies (Harms et al., 2010). Neurotypical FER ability improves with age in childhood and into adolescence (Durand et al., 2007). However, as children and adults with ASC did not differ in their FER ability (O’Connor et al., 2005), and as there was no correlation between age and FER performance in ASC (Gepner et al., 2001), FER ability in ASC may reach its peak much earlier on (Tanaka et al., 2012). Second, autistic individuals may range from above average IQ and limited verbal difficulties through to low IQ and profound language difficulties (Burack & Volkmar, 1992). As the inclusion of different presentations of ASC varies both across and within FER studies, this could determine whether an autistic individual is impaired at FER (Harms et al., 2010).

Third, mixed results could be attributed to methodological differences between studies (Tanaka et al., 2012). FER tasks involving matching emotional expressions could be less sensitive to detecting ASC deficits as they may allow for autistic individuals to use compensatory strategies such as only processing the stimuli’s surface characteristics without fully understanding the emotion displayed (Hariri et al., 2000; Teunisse & de Gelder, 2001). By contrast, although forced-choice labelling tasks may allow for the guessing of correct answers, which is particularly an issue in studies that had only two label options (e.g. Baron-Cohen et al., 1997), labelling tasks that require the individual to freely generate their answer may be affected by verbal ability and thus may be representative of language rather than FER ability (Uljarevic & Hamilton, 2013). Fourth, the variables on which the ASC group was matched to controls could contribute to inconsistent results as some variables may mask or enhance FER performance (Tanaka et al., 2012). As cited in Tanaka et al. (2012), studies have shown FER deficits in ASC when participants were matched on non-verbal intelligence, yet no ASC deficit when matched on verbal intelligence (Fein et al., 1992; Ozonoff et al., 1990).

Research has recently concluded that the strongest explanation for the inconsistency in FER results is the alexithymia hypothesis (Kinnaird et al., 2019; Poquérusse et al., 2018). Alexithymia is a subclinical phenomenon characterised by difficulties in recognising and describing one’s own emotional state (Nemiah et al., 1976). Because it involves having problems with understanding what bodily or emotional sensations one experiences, communicating this to others, and having a cognitive style that is focussed on the external details of life rather than one’s inner experience (Bagby et al., 1994), it often leads to more distant interpersonal relationships (Kafetsios & Hess, 2019). While the prevalence of alexithymia in the general population is estimated at 10% (Linden et al., 1995), 40%–65% of individuals with ASC meet its diagnostic criteria (Griffin et al., 2016). It is important to note, however, that alexithymia and ASC are distinct constructs as alexithymia is not necessary or sufficient to receive an ASC diagnosis and vice versa (Cook et al., 2013). The alexithymia hypothesis states that impaired FER is not a core feature of ASC, but instead represents a feature of their co-occurring alexithymia (Bird & Cook, 2013).

An increasing number of studies have supported the alexithymia hypothesis (Kinnaird et al., 2019). Alexithymia, independent of ASC, is strongly associated with a deficit in FER (Grynberg et al., 2012). Neurotypical individuals who score highly on the 20-item Toronto Alexithymia Scale (TAS-20; Bagby et al., 1994), the most reliable and widely used self-report tool for measuring alexithymia (Prkachin et al., 2009), are poorer at FER than those with low alexithymia scores (e.g. Lane et al., 2000; Swart et al., 2009). Recent studies have found that co-occurring alexithymia, and not ASC diagnosis, predicts poor FER ability in autistic individuals (Bird & Cook, 2013; Kinnaird et al., 2019). Cook et al. (2013) found no differences between adults with ASC and neurotypical controls in their ability to identify which of the six basic emotions was displayed on the faces of morphed stimuli. As there was a negative correlation between alexithymia level and FER ability, it was concluded that the groups did not differ because there was an equal distribution of those with high alexithymia in each group. With no correlation between ASC severity and FER, these findings support the alexithymia hypothesis. Similar results have been found in a younger sample, as autistic adolescents who were higher in alexithymia had more parent-reported emotional difficulties and performed worse on FER than those low in alexithymia (Milosavljevic et al., 2016). Importantly, participants’ ASC severity scores were unrelated to FER (Milosavljevic et al., 2016). Interestingly, support for the alexithymia hypothesis is not restricted to only explaining emotional deficits regarding faces as autistic individuals with high levels of alexithymia, and not those with low alexithymia, were impaired at recognising emotion from voices (Heaton et al., 2012) and from music (Allen et al., 2013). It is important to note, however, that alexithymia may not explain all emotional symptoms associated with ASC, as a recent study found that it was autistic adults’ autistic traits, and not their level of alexithymia, that predicted reduced eye fixation on emotional facial stimuli (Stephenson et al., 2019).

Yet most studies that support the alexithymia hypothesis have fundamental limitations. First, most studies have used FER tasks comprised of facial stimuli that lack ecological validity. In the real world, people recognise briefly presented multimodal emotional expressions, meaning that facial, vocal and bodily cues are simultaneously displayed (Loth et al., 2018; Schlegel & Scherer, 2016). As most tasks in these studies used Ekman and Friesen’s (1976) grayscale static photographs of actors posing exaggerated prototypical emotions (e.g. Cook et al., 2013; Milosavljevic et al., 2016; J. D. Parker et al., 1993), performance on them cannot fully reflect how individuals process facial expressions in daily life. Second, almost all studies supporting the alexithymia hypothesis have only measured the six basic emotions which are not fully representative of the range of complex emotions perceived in life (e.g. Cook et al., 2013; Heaton et al., 2012). Although one study supported the alexithymia hypothesis using the Reading the Mind in the Eyes Test (RMET; Baron-Cohen et al., 2001), which tests a range of complex emotions identified from the eye region, (Oakley et al., 2016), the RMET’s stimuli are still only static, grayscale photographs of eye regions that are identified without the holistic context of all the facial features, as one would do in real life (Fertuck et al., 2009). Third, these studies typically have small samples. With samples as small as 16 (Cook et al., 2013) or 13 participants (Bird et al., 2011), studies like these may lack the statistical power to detect differences in FER ability between ASC and controls. Fourth, studies investigating the alexithymia hypothesis typically do not have FER tests that measure how long participants take to identify emotions (e.g. Cook et al., 2013; Milosavljevic et al., 2016). Although one recent study has measured RT of FER within the context of alexithymia and found that alexithymia was not a significant predictor of how long those with and without ASC took to identify emotions, this was only measured for a small subset of emotions (fear, joy, anger, neutral; Stephenson et al., 2019). Including RT for a wider range of emotions could help determine whether alexithymia does predict slower processing speed of emotions (Ketelaars et al., 2016).

Finally, perhaps one of the most important limitations is that all but one study examining the alexithymia hypothesis in ASC has consisted of all-male (e.g. Bird et al., 2010) or predominantly male participants with ASC (e.g. Bird et al., 2011; Cook et al., 2013; Oakley et al., 2016). Because ASC is approximately three times more prevalent in males than females (Loomes et al., 2017), most other findings on the mechanisms that underlie ASC symptoms are also based on samples dominated by males (e.g. Peterson et al., 2016). Thus, one must be cautious about automatically assuming findings apply to autistic females, due to sex differences within ASC that may reflect a different ASC phenotype for females (Ketelaars et al., 2016; Rivet & Matson, 2011).).

Research on the FER ability in females has been as inconsistent as studies that included predominantly autistic male samples (Uljarevic & Hamilton, 2013). One study with a sufficient number of participants did find an FER deficit in autistic females (Lai et al., 2012), while another did not (Ketelaars et al., 2016). Ketelaars et al. (2016) measured autistic female adults’ and matched controls’ levels of alexithymia, their ASC symptomology, and had them label facial emotions of varying intensity. No differences in FER ability were found between the ASC and control groups, but females from either group with higher levels of alexithymia were significantly worse at recognising subtle low-intensity emotions compared to those with lower alexithymia. As ASC symptomatology was not associated with FER ability, this supports the alexithymia hypothesis for autistic females. However, this study shares several of the common methodological limitations among studies investigating the alexithymia hypothesis. Only grayscale images of the basic emotions were used in the FER test, and RT was not measured to provide more conclusive results. Further research in autistic females that addresses these limitations is therefore vital.

The present study aimed to address these limitations by using more ecologically valid methodology and a large all-female sample to determine whether the alexithymia hypothesis applies to autistic females. It aimed to investigate whether higher alexithymia levels, and not ASC severity, is associated with poorer FER ability in autistic females, and which sub-categories of alexithymia, if any at all, are specifically associated with FER. Female adults with ASC self-reported their level of alexithymia and ASC severity. They also completed the Geneva Emotion Recognition Test–Short (GERT-S; Schlegel & Scherer, 2016): an FER test that consists of video clips of actors multimodally portraying a range of complex emotions in full colour that measures both accuracy and RT. The hypotheses were (1) there will be a negative correlation between alexithymia and FER ability: autistic females who report higher levels of alexithymia (TAS-20 score) will have poorer FER ability (lower percentage of correct responses in the GERT-S and slower average RT) than those with lower alexithymia levels and (2) there will be no association between ASC severity (Autism Spectrum Quotient total score; Baron-Cohen et al., 2001) and FER ability.

## Methods

### Participants

A total of 217 females participated in the study. As 92 started the study but did not complete it, this left 125 participants with complete data sets. Clinical diagnosis was determined by having participants select online whether they have a clinical diagnosis of ASC, self-identify as having ASC, or neither self-identify nor have a clinical diagnosis; 42 participants self-identified as having ASC, and 1 participant neither self-identified nor was diagnosed with ASC. These participants were excluded from the core analysis in order to ensure a ‘valid’ ASC sample. Thus, core analyses were conducted on 83 females who stated they have a clinical diagnosis of ASC. In this final sample (*n* = 83), participants were aged between 19 and 65 years, with the mean age of 38.5 years (standard deviation (SD) = 11.72 years). All participants volunteered to take part by following the study’s online Qualtrics link (Qualtrics, 2019) advertised in various autism groups or charity pages on Facebook (e.g. ‘Aspire: The Female Autism Network’), as well as through Twitter. The only participant exclusion criteria (noted on the advertisement) was being male, under the age of 18 years and/or not having or self-identifying as having a diagnosis of an ASC.

### Materials

#### Autism spectrum disorder severity

Severity of autistic traits was measured using the Autism Spectrum Quotient (AQ; Baron-Cohen et al., 2001). The AQ is a 50-item self-administered questionnaire that measures level of traits associated with autism spectrum disorder (ASD). It is divided into five subscales consisting of 10 items, including Social Skill, Attention Switching, Attention to Detail, Communication and Imagination. Participants’ record whether they ‘strongly agree’, ‘agree’, ‘disagree’, or ‘strongly disagree’ with each item. The maximum score is 50, the minimum is 0. Higher AQ scores reflect greater number of ASC traits, with a cut-off score of 32 and above reflecting a clinically significant level of autistic traits. The AQ has good test–retest reliability (*r* = 0.79; Hoekstra et al., 2008) and has been validated as a reliable measure of ASC severity in the autistic population (Broadbent et al., 2013). In addition, it has often been used to measure ASC severity in previous studies investigating the alexithymia hypothesis in ASC, justified by the correlation between total AQ score and ASC diagnosis (e.g. Cook et al., 2013; Shah et al., 2016).

#### Alexithymia

Levels of alexithymia were measured using the 20-item TAS-20; Bagby et al., 1994), a self-administered instrument assessing the degree of difficulty one has in recognising and describing their own emotional state. It has three subscales including Difficulty Identifying Feelings, Difficulty Describing Feelings and Externally Oriented Thinking. Items are scored with a 5-point Likert-type scale (5 = *strongly agree*, 4 = *agree*, 3 = *neither agree* or *disagree*, 2 = *disagree*, 1 = *strongly disagree*), with 5 of the 20 items being reverse scored. With a minimum score of 20 and a maximum of 100, scoring 51 or less indicates no alexithymia; between 52 and 60 indicates possible alexithymia; and above 61 indicates alexithymia. The TAS-20 is viewed by Berthoz and Hill (2005) as a validated and reliable psychometric instrument for identifying alexithymia in the autistic population, with high test–retest reliability (*r* = 0.77), convergent validity (*r* = 0.62) and internal consistency (Cronbach’s α = 0.81; Bagby et al., 1994).

#### FER ability

**Figure 1.**
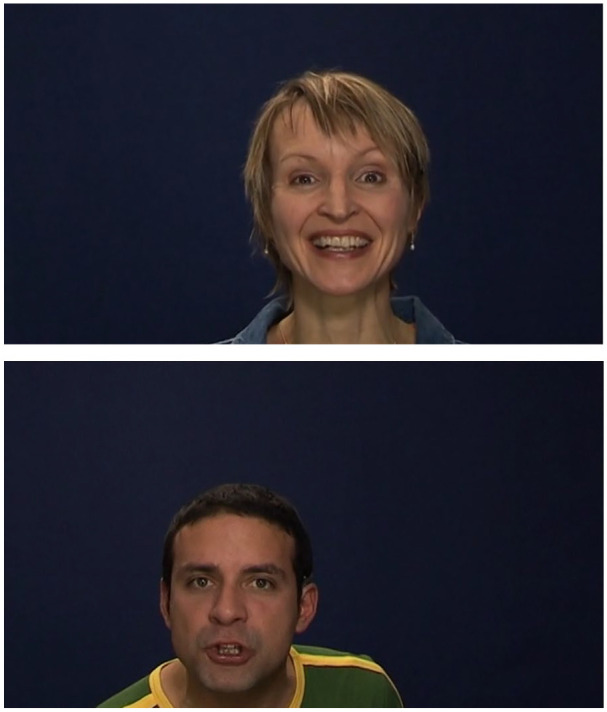
Screenshots of example videos from the GERT-S with actors expressing joy (top: female) and anger (bottom: male).

FER was assessed with the computer-administered GERT-S (Schlegel & Scherer, 2016), which has demonstrated good construct validity and internal consistency (Schlegel & Scherer, 2016). It consists of 42 video clips, each lasting 1–3 s, of five male and female professional French-Swiss actors expressing 14 different emotions: pride, anger, joy, irritation, amusement, disgust, pleasure, sadness, relief, despair, interest, fear, surprise and anxiety. The clips were selected from the Geneva Multimodal Emotion Portrayals database and had increased authenticity as they were recorded as part of a continuous interaction between a director and actor role-playing a real-life scenario (Bänziger et al., 2012). Each clip presented an actor’s upper torso and face portraying an emotion through their facial expression, gestures and voice as actors pronounced a phrase that had no semantic meaning but conveyed an emotional tone (see Figure 1 for screenshots of example video clips); 12 of the emotions were selected because they covered the four core emotional categories of the combinations of high/low arousal and positive/negative valence as introduced by Bänziger et al. (2012), while disgust and surprise were included as these are traditionally assessed in most FER tests.

**Figure 2.**
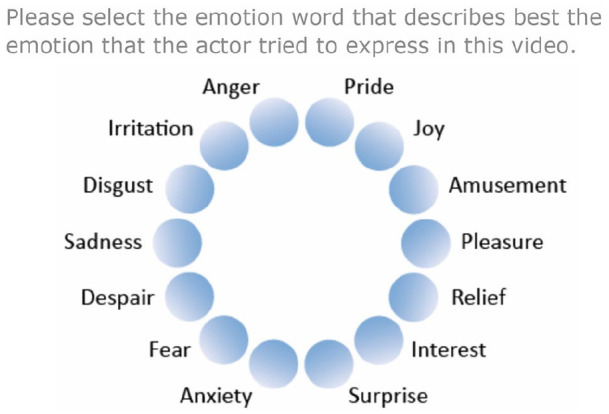
Emotion wheel; the GERT-S response format. The 14 emotions are arranged in a circle to facilitate participants’ ability to orient among the options (Schlegel & Scherer, 2016).

The test presented participants with simple instructions, followed by a short definition of each of the 14 emotions. For each trial, participants clicked inside the video to play it and were then presented with an emotion wheel to select the correct emotion the actor in the video was intending to portray (see Figure 2 for GERT-S response format). The 14 emotion options were arranged in a circle to facilitate participants’ ability to orient among the options. No choice feedback was provided at the end of each trial or the test. Participants first completed two practice trials, followed by the remaining 42 trials. The time taken to choose the correct emotion, and the number of times participants clicked in the emotion wheel, was not restricted. However, each video could only be played once. The GERT-S is typically completed within 10 min and is scored by allocating 1 point for each correctly identified trial and 0 for incorrect trials. The test also recorded RT from when each video ended to when participants clicked on their final choice in the emotion wheel.

### Design

The study used a correlational design with the variables: total AQ score (and scores for each of the five AQ subscales), total TAS-20 score (and scores for its three subscales), as well as GERT-S score (for both percentage accuracy and average RT).

### Procedure

The study was presented on an online Qualtrics survey and participants followed a Qualtrics link to complete it on a computer/laptop via a web browser. The opening screen presented information explaining what participants need to do. All participants gave full written consent to participate and were provided with a randomly generated 5-digit unique ID to withdraw their data if necessary. Participants first completed the AQ, followed by the TAS-20, by selecting the response that best describes how strongly they felt each statement applies to them. They then completed the GERT-S and were instructed that they will watch each video once with headphones; that they will not be able to understand what the actors are saying; and that they are to select the emotion word that best describes the emotion the actor wanted to express. Participants were finally debriefed about the aims of the study. Total time taken to complete the study was approximately 30 min, and participants were given the opportunity to be rewarded for their time by following an alternative Qualtrics link to enter a prize draw to win one of the 12 £25 Amazon Vouchers, funded by Newcastle University.

### Results

#### AQ and TAS-20 scores

Participants scored between 11 and 49 on the AQ (*M* = 38.3, *SD* = 7.45) and 84.3% of the sample scored above clinical cut-off (AQ ⩾ 32). TAS-20 scores ranged from 32 to 88 (*M* = 65.2, *SD* = 11.4). According to the thresholds of the TAS-20, 72.3% of participants could be identified as having alexithymia (TAS-20 ⩾ 61), 15.7% had borderline alexithymia (TAS-20: 52–60), and 12% had no alexithymia (TAS-20 ⩽ 51). However, given that small differences in scores across thresholds could result in inaccurate group allocation and that these group sizes were unequal, all further analyses treated TAS-20 and AQ scores as being on a continuous scale, with participants having higher or lower levels of alexithymia and ASC severity.

#### GERT-S performance

**Figure 3.**
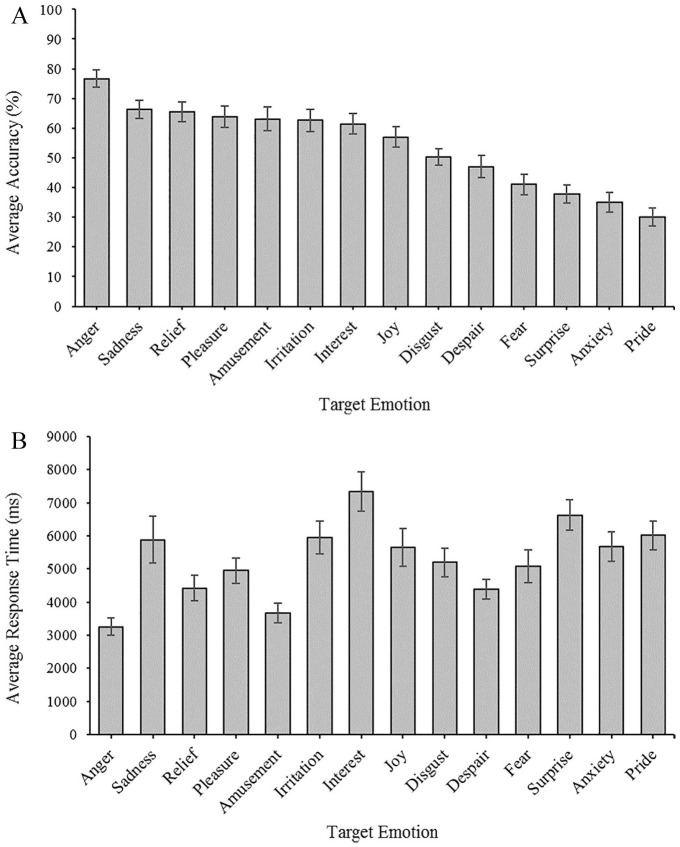
Average accuracy and average response time on GERT-S.

Figure 3 shows participants’ average accuracy and response time for each individual emotion on the GERT-S. To measure participants’ FER ability, the total number of correct answers on the GERT-S for each participant (accuracy), and the average time taken to correctly respond to each trial (RT), was recorded. Overall, there were two outliers for average RT. Participants’ average accuracy was 54.1% (*SD* = 14.5%). While the average RT for a trial was 4400 ms (*SD* = 2143) for correct responses, paired samples *t*-tests showed that participants took significantly longer to identify the emotion on trials they got incorrect (*M* = 6482 ms, *SD* = 3027 ms), compared to those they got correct (*M* = 4401 ms, *SD* = 2144 ms), *t*(82) = –9.59, *p* < 0.001.

#### Examining the alexithymia hypothesis

To assess whether the alexithymia hypothesis does apply to female adults diagnosed with ASC, Spearman’s rho correlations were conducted between alexithymia (TAS-20 total score and subscales) and FER ability (GERT-S average percentage accuracy and RT) and between ASC severity (AQ total score and subscales) and FER ability. A non-parametric test was implemented as all variables were checked for normality using Shapiro–Wilk and were not normally distributed. Spearman’s rho correlation indicated there was a significant positive relationship between total TAS-20 and total AQ (*r_s_*(83) = 0.35, *p* = 0.001), thus greater ASC severity was related to higher alexithymia levels. Consistent with the alexithymia hypothesis, while there was no relationship between total AQ and accuracy on the GERT-S (*r_s_*(83) = –0.16, *p* = 0.16), there was a significant negative correlation between total TAS-20 score and GERT-S accuracy (*r_s_*(83) = –0.27, *p* = 0.012). Therefore, higher alexithymia, not ASC severity, was associated with poorer FER accuracy.

Correlations between the subscales of the AQ and TAS-20 with GERT-S performance revealed which specific components of alexithymia (and ASC) were associated with FER ability. As summarised in Table 1, higher alexithymia scores on both Difficulty Identifying Feelings and Externally Oriented Thinking were significantly associated with poorer FER accuracy, while Difficulty Describing Feelings was unrelated to FER. As Table 2 shows, the only AQ subscale that was significantly correlated to FER accuracy was Communication. No further components of ASC were associated with FER.

**Table 1.** Spearman’s rho correlations of alexithymia levels (TAS-20) and FER ability (GERT-S) in females diagnosed with ASC (*n* = 83).

	GERT-S total accuracy		GERT-S average RT	
	Spearman’s rho (*r_s_*)	*p*	Spearman’s rho (*r_s_*)	*p*
TAS-20 Total	−0.27*	0.012	−0.15	0.17
TAS-20 Difficulty Describing Feelings	−0.12	0.30	−0.16	0.15
TAS-20 Difficulty Identifying Feelings	−0.29*	0.007	−0.15	0.13
TAS-20 Externally Oriented Thinking	−0.27*	0.01	−0.09	0.44

GERT-S: Geneva Emotion Recognition Test–Short; RT: response time; TAS-20: Toronto Alexithymia Scale.

* *p* < 0.05.

**Table 2.** Spearman’s rho correlations of ASC severity (AQ) and FER ability (GERT-S) in females diagnosed with ASC (*n* = 83).

	GERT-S total accuracy		GERT-S average RT	
	Spearman’s rho (*r_s_*)	*p*	Spearman’s rho (*r_s_*)	*p*
AQ Total	−0.16	0.16	0.055	0.62
AQ Social Skills	−0.091	0.41	0.13	0.25
AQ Attention Switching	−0.005	0.96	0.003	0.99
AQ Attention to Detail	0.048	0.67	0.24*	0.03
AQ Communication	−0.22*	0.044	0.004	0.97
AQ Imagination	−0.21	0.057	–0.12	0.30

GERT-S: Geneva Emotion Recognition Test-Short; RT: response time; AQ: Autism Spectrum Quotient.

* *p* < 0.05.

However, as ASC severity could account for a significant proportion of the unique variance when alexithymia is taken into account, further hierarchical regression analyses were conducted to determine whether TAS-20 predicts FER accuracy above and beyond ASC severity. With GERT-S average percentage accuracy as the outcome variable, total AQ was entered into the first step of the model and total TAS-20 was entered into the second step. While total AQ was not a significant predictor of FER accuracy (*F*(1, 81) = 0.390, *p* = 0.534, *R*^2^ = 0.005), total TAS-20 was a significant predictor of FER accuracy (*F*(2, 80) = 4.722, *p* = 0.012, *R*^2^ = 0.106). Alexithymia significantly improved the model and significantly increased the variance accounted for by 10.1%.

Considering RT on the GERT-S, as shown in both Tables 1 and 2, while no significant correlations were observed between TAS-20 scores and GERT-S average RT, the only significant correlation between AQ and RT was between the AQ subscale of Attention to Detail and GERT-S average RT. Thus, those who took longer on average to correctly identify an emotion had a higher attention to detail. A multiple regression analysis was conducted with GERT-S average RT as the outcome variable, with total AQ entered at the first step of the model, and total TAS-20 entered at the second step. Both total AQ (*F*(1, 81) = 0.273, *p* = 0.603, *R*^2^ = 0.003) and total TAS-20 (*F*(2, 80) = 1.459, *p* = 0.239, *R*^2^ = 0.035) were not significant predictors of GERT-S average RT. Therefore, alexithymia levels (and ASC severity) were not associated with the speed of emotion processing in autistic females.

These analyses were also conducted on a sample (*n* = 117) that included females with a clinically significant level of autistic traits (total AQ ⩾ 32) who self-identified as having ASC. While the pattern of results was the same for this sample regarding alexithymia and FER accuracy, as well as RT findings, results differed in that total AQ, AQ Communication and AQ Imagination significantly correlated with FER accuracy (see Appendix 1 for full analyses). However, multiple regression analyses revealed that ASC severity was not a significant predictor of GERT-S accuracy (*F*(1, 123) = 1.344, *p* = 0.249, *r*^2^ = 0.011), while alexithymia was (*F*(2, 122 = 5.881, *p* = 0.004, *r*^2^ = 0.088).

## Discussion

By addressing methodological limitations of previous research, this study aimed to determine whether the alexithymia hypothesis applies to autistic females; to identify which sub-category of alexithymia, if any, is associated with FER ability; and to explore prevalence of alexithymia in autistic females. As hypothesised, higher levels of alexithymia and not ASC severity were associated with poorer FER accuracy. Having greater difficulty in identifying one’s own feelings and externally oriented thinking was related to poorer FER accuracy. Difficulty in describing feelings was the only sub-category of alexithymia not associated with FER accuracy. However, the finding that participants higher in alexithymia were not slower at FER than those lower in alexithymia did not support the original hypothesis. Thus, the alexithymia hypothesis applied to autistic females’ accuracy in FER, but not to their speed of emotion processing.

This study provided a valuable indication of the prevalence of alexithymia in females with ASC. The finding that the large majority of autistic females in this study could be identified as having high alexithymia is consistent with previous findings of how rates of alexithymia tend to be significantly higher in ASC compared to neurotypical samples (Heaton et al., 2012; Honkalampi et al., 2000). However, prevalence in this sample was higher than in other reported autistic samples. While almost three quarters of participants in this study reported high alexithymia, the prevalence of alexithymia has previously been reported as between 40% and 65% in male (Hill et al., 2004; Mul et al., 2018) and female autistic samples (Ketelaars et al., 2016). One explanation for why the prevalence was so high among this sample could be selection bias. As the study was advertised as investigating emotion recognition in ASC, it is possible that autistic women who struggle more with FER (and thus those higher in alexithymia) were more likely to self-select. However, there is currently little evidence to explain why the prevalence of alexithymia is generally higher in the autistic population. While this could be because alexithymia and ASC both have shared difficulties in mentalising (Kinnaird et al., 2019), Bird and Cook (2013) have speculated that it may be the result of a genetic vulnerability to developing atypical neural connectivity. Individuals with poorer neural connectivity within social cognition networks may develop ‘pure’ ASC, and those with connectivity issues among emotional networks may develop ‘pure’ alexithymia, but it is more likely for individuals to have poorer connectivity across both networks, leading to more frequent co-occurrence of alexithymia and ASC (Bird & Cook, 2013). This hypothesis warrants further investigation.

The main and most compelling finding was that autistic females’ level of alexithymia, and not ASC severity, was associated with FER ability. Those with greater difficulty in recognising their own emotions were less accurate at recognising the emotions of others. This is in line with research that supports a general FER deficit in alexithymia regardless of ASC (Grynberg et al., 2012; Lane et al., 1996) and adds to the few but growing number of studies that have supported this alexithymia hypothesis in ASC (Allen et al., 2013; Cook et al., 2013; Heaton et al., 2012; Ketelaars et al., 2016; Milosavljevic et al., 2016). Ketelaars et al. (2016) similarly found that it was alexithymia in autistic females that predicted FER of low-intensity emotions. The present study builds upon this with a larger sample of autistic women. While previous studies were only able to demonstrate that alexithymia is associated with recognition of static photographs of basic emotions, the present study has supported the alexithymia hypothesis with greater ecological validity, suggesting autistic women who are higher in alexithymia are poorer at recognising multimodal displays of a range of emotions in the way they typically would be perceived in real life.

It is possible perceiving and experiencing emotions share the same underlying neural networks (Wingbermühle et al., 2012), as consistent with the shared network model of social cognition (Preston & De Waal, 2002). When perceiving another’s emotional expression, a neural simulation or representation of this emotion is directly experienced (Preston & De Waal, 2002). This enables individuals to automatically use their experience of the emotion they perceive to help them to recognise it. Damage to neural structures that underlie the experience of a certain emotion can lead to an impaired ability to recognise it in others (Adolphs et al., 2005; Heberlein & Atkinson, 2009), and neurotypical individuals with high alexithymia (who also had impaired FER) show weaker neural responses in regions that are typically activated when processing various emotions (Grynberg et al., 2012; Ihme et al., 2014a).

The present study also indicated that FER ability in autistic women was associated with two out of three core components of alexithymia. While greater issues in identifying one’s own emotions and externally oriented thinking were related to poorer FER accuracy, having difficulty describing one’s own feelings was not. Since no previous study involving autistic participants appears to have assessed which components of alexithymia are associated with FER, the current study may have been the first to demonstrate this. Interestingly, this differs to research on non-clinical samples. Ihme et al. (2014a) and Swart et al. (2009) found that difficulty describing feelings was most strongly associated with basic FER, perhaps because this ability, like attributing a label to a facial expression, involves semantically processing emotional information (Ihme et al., 2014a). Difficulty identifying feelings was instead associated with the automatic neural processing of briefly presented emotional faces (Ihme et al., 2014a). Contrasting results in the present study may be due to the realistic nature of the FER test. Because the GERT-S presented complex and multimodal emotions, accurate performance may have relied on automatic emotional processing. Although participants did label emotional expressions in this task, being able to describe their feelings may not have been relevant in facilitating performance. The finding that externally oriented thinking was related to poorer FER accuracy in autistic women coincides with studies showing that higher scores on this cognitive style correlated with poorer recognition of emotion from faces (Prkachin et al., 2009) and music (Taruffi et al., 2017).

The finding that participants with higher autistic traits in communication had poorer FER accuracy was somewhat surprising considering that difficulty describing feelings was not associated with FER. However, it is possible that having poorer FER, associated with alexithymia, impacts one’s communication ability. If an autistic female struggles to accurately identify the emotion on another’s face, they may not understand the true meaning of another’s words, how to respond to them appropriately or, for example, whether another person is indicating to them that they are talking excessively. All of these non-verbal cues that rely on accurate FER are vital for effective communication (Kothari et al., 2013).

The finding that alexithymia was not associated with the speed of emotion processing is of interest. Most studies that have previously investigated the alexithymia hypothesis in ASC have not measured RT of FER (e.g. Cook et al., 2013; Ketelaars et al., 2016). Research investigating neurotypical participants has found that individuals with greater difficulty in describing their feelings were slower at recognising the negative basic emotions (Ihme et al., 2014b) or struggled to identify them when they were presented very briefly (P. D. Parker et al., 2005). Such research concluded those high in alexithymia need more time to recognise an emotion perhaps because they do not generate automatic representations of another’s emotion (Grynberg et al., 2012), or because they use processing strategies that are not as efficient as those used by individuals with low alexithymia (Vermeulen et al., 2008). The finding in the current study is more in line with those that have similarly shown no relation between RT of FER and alexithymia (Mériau et al., 2006; Vermeulen et al., 2008). Studies that have shown slower FER in ASC compared to controls may not reflect higher levels of alexithymia in the autistic sample (Bal et al., 2010), but could reflect an overall slower processing speed in ASC in general (Travers et al., 2014).

Overall, it is important to discuss the secondary analysis in our study that demonstrated that when those who self-identified as having an ASC were included into the sample with women with a formal diagnosis of ASC, ASC severity (as well as alexithymia) was significantly correlated with FER accuracy. Although ASC severity did not significantly predict FER accuracy for the formally diagnosed sample, it is recommended that future studies continue to investigate the alexithymia hypothesis in female samples to more significantly determine whether ASC does have any association with FER ability.

Although this study has addressed several limitations of previous similar studies, it is not without limitations of its own. Perhaps the largest limitation concerns the sample. Although relying on convenience sampling meant that we were able to sample a large amount of participants, we were unable to fully verify that those who stated they had a clinical diagnosis of ASC actually did. Given that more extensive demographic information about the sample was not gathered (e.g. education), it may also be difficult to know who these results can generalise to. One further limitation is that the AQ was used as a measure of participants’ ASC severity. Although the AQ is typically used for this purpose in similar research (e.g. Cook et al., 2013), it is not a diagnostic tool and thus higher scores on it do not always reflect the presence of autism. While all females in the core analysis did have an ASC diagnosis, the key finding that poorer FER was not associated with higher scores on the AQ, although indicative may not conclusively suggest that it is not part of autism. This leads to the third limitation: because this study did not include a matched sample of neurotypical females, it was unable to determine whether there is any difference between females with or without ASC in FER ability. Future research would benefit from the inclusion of control groups to determine whether it is alexithymia, regardless of diagnosis, that predicts FER ability. Furthermore, although the TAS-20 is a fast instrument to complete and administer, it may not have estimated accurate levels of alexithymia in this particular sample. Given that individuals high in alexithymia and those with ASC may struggle with self-insight and identifying or describing aspects about themselves (Griffinet al., 2016), self-report measures require a basic level of emotional awareness, and thus, levels of alexithymia (and ASC) may have been underestimated (Bird & Cook, 2013). Future research should be aware of this issue and consider exploring ways to measure alexithymia in a way that does not require self-insight (Bird & Cook, 2013). Finally, while this study used an FER test with significantly increased ecological validity compared to static photographs of just a subset of emotions, identifying emotions from short video clips with actors expressing incomprehensible phrases is still not how humans process emotions in everyday life. Studies could benefit from implementing FER tests that are even closer to real life, perhaps by having participants recognise emotions in vivo rather than on a computer screen.

## Conclusion

Overall, by counteracting some of the limitations of previous research, this study has provided evidence for how the alexithymia hypothesis applies to females diagnosed with ASC. Alexithymia in autistic women, specifically in terms of difficulty identifying their own emotions and externally oriented thinking, but not their ASC, was associated with FER ability. However, this was only for FER accuracy, as speed of emotion processing was unrelated to both ASC and alexithymia.

To our knowledge, the present study was the first to implement an ecologically valid FER test assessing a range of complex emotions and RT when investigating the alexithymia hypothesis in ASC. Future studies could use similar FER tests to explore alexithymia and FER ability in autistic males. Furthermore, while the prevalence of alexithymia is elevated in the autistic population (e.g. Brewer et al., 2015), there is limited evidence explaining why the two conditions tend to co-occur. It would be useful to investigate the underlying mechanisms of alexithymia and why this overlaps with ASC. Finally, it is important to explore whether the alexithymia hypothesis explains the emotional impairments associated with conditions other than ASC. For example, FER ability in anorexia nervosa may also be explained by higher prevalence of alexithymia in this population (Zonnevijlle-Bendek et al., 2002). Studies could explore whether this applies to other clinical conditions, particularly those characterised by a high prevalence of alexithymia and inconsistent FER results, such as schizophrenia (Cedro et al., 2001; Kring & Elis, 2013).

## Appendix 1

Data analysis including females with AQ ⩾32 who self-identified as having ASC.

**Table A1.** Spearman’s rho correlations of alexithymia (TAS-20) and FER ability (GERT-S) in diagnosed autistic females and those scoring AQ ⩾ 32 who self-identified as having ASC (*n* = 117).

	GERT-S total accuracy		GERT-S average RT	
	Spearman’s rho (*r_s_*)	*p*	Spearman’s rho (*r_s_*)	*p*
TAS-20 Total	−0.31*	0.001	−0.12	0.20
TAS-20 Difficulty Describing Feelings	−0.17	0.073	−0.16	0.079
TAS-20 Difficulty Identifying Feelings	−0.28*	0.002	−0.12	0.19
TAS-20 Externally Oriented Thinking	−0.31*	0.001	−0.039	0.68

GERT-S: Geneva Emotion Recognition Test-Short; RT: response time; TAS-20: Toronto Alexithymia Scale.

* *p* < 0.05.

**Table A2.** Spearman’s rho correlations of ASC severity (TAS-20) and FER ability (GERT-S) in diagnosed autistic females and those scoring AQ ⩾ 32 who self-identified as having ASC (*n* = 117).

	GERT-S total accuracy		GERT-S average RT	
	Spearman’s rho (*r_s_*)	*p*	Spearman’s rho (*r_s_*)	*p*
AQ total	−0.22*	0.020	0.079	0.40
AQ social skills	−0.13	0.16	0.11	0.25
AQ attention switching	−0.068	0.47	0.072	0.44
AQ attention to detail	0.077	0.41	0.14	0.13
AQ communication	−0.26*	0.005	0.003	0.98
AQ imagination	−0.24*	0.010	−0.022	0.82

GERT-S: Geneva Emotion Recognition Test-Short; RT: response time; AQ: Autism Spectrum Quotient.

* *p* < 0.05.

## References

[bibr1-1362361320932727] Adolphs R. Gosselin F. Buchanan T. W. Tranel D. Schyns P. Damasio A. R. (2005). A mechanism for impaired fear recognition after amygdala damage. Nature, 433(7021), 68–72. 15635411 10.1038/nature03086

[bibr2-1362361320932727] Adolphs R. Sears L. Piven J. (2001). Abnormal processing of social information from faces in autism. Journal of Cognitive Neuroscience, 13(2), 232–240. 11244548 10.1162/089892901564289

[bibr3-1362361320932727] Allen R. Davis R. Hill E. (2013). The effects of autism and alexithymia on physiological and verbal responsiveness to music. Journal of Autism and Developmental Disorders, 43(2), 432–444. 22752845 10.1007/s10803-012-1587-8

[bibr4-1362361320932727] American Psychiatric Association. (2013). Diagnostic and statistical manual of mental disorders: DSM-5.

[bibr5-1362361320932727] Ashwin C. Chapman E. Colle L. Baron-Cohen S. (2006). Impaired recognition of negative basic emotions in autism: A test of the amygdala theory. Social Neuroscience, 1(3–4), 349–363. 18633799 10.1080/17470910601040772

[bibr6-1362361320932727] Bagby R. M. Parker J. D. Taylor G. J. (1994). The twenty-item Toronto Alexithymia Scale – I. Item selection and cross-validation of the factor structure. Journal of Psychosomatic Research, 38(1), 23–32. 8126686 10.1016/0022-3999(94)90005-1

[bibr7-1362361320932727] Baio J. (2014). Prevalence of autism spectrum disorder among children aged 8 years – Autism and developmental disabilities monitoring network, 11 sites, United States, 2010. MMWR Surveillance Summaries, 63, 1–21. 24670961

[bibr8-1362361320932727] Bal E. Harden E. Lamb D. Van Hecke A. V. Denver J. W. Porges S. W. (2010). Emotion recognition in children with autism spectrum disorders: Relations to eye gaze and autonomic state. Journal of Autism and Developmental Disorders, 40(3), 358–370. 19885725 10.1007/s10803-009-0884-3

[bibr9-1362361320932727] Bänziger T. Mortillaro M. Scherer K. R. (2012). Introducing the Geneva multimodal expression corpus for experimental research on emotion perception. Emotion, 12(5), 1161–1179. 22081890 10.1037/a0025827

[bibr10-1362361320932727] Baron-Cohen S. Jolliffe T. Mortimore C. Robertson M. (1997). Another advanced test of theory of mind: Evidence from very high functioning adults with autism or Asperger syndrome. Journal of Child Psychology and Psychiatry, 38(7), 813–822. 9363580 10.1111/j.1469-7610.1997.tb01599.x

[bibr11-1362361320932727] Baron-Cohen S. Scott F. J. Allison C. Williams J. Bolton P. Matthews F. E. Brayne C. (2009). Prevalence of autism-spectrum conditions: UK school-based population study. The British Journal of Psychiatry, 194(6), 500–509. 19478287 10.1192/bjp.bp.108.059345

[bibr12-1362361320932727] Baron-Cohen S. Wheelwright S. Skinner R. Martin J. Clubley E. (2001). The autism-spectrum quotient (AQ): Evidence from Asperger syndrome/high-functioning autism, males and females, scientists and mathematicians. Journal of Autism and Developmental Disorders, 31(1), 5–17. 11439754 10.1023/a:1005653411471

[bibr13-1362361320932727] Berthoz S. Hill E. L. (2005). The validity of using self-reports to assess emotion regulation abilities in adults with autism spectrum disorder. European Psychiatry, 20(3), 291–298. 15935431 10.1016/j.eurpsy.2004.06.013

[bibr14-1362361320932727] Bird G. Cook R. (2013). Mixed emotions: The contribution of alexithymia to the emotional symptoms of autism. Translational Psychiatry, 3(7), 1–8. 10.1038/tp.2013.61 PMC3731793 23880881

[bibr15-1362361320932727] Bird G. Press C. Richardson D. C. (2011). The role of alexithymia in reduced eye-fixation in autism spectrum conditions. Journal of Autism and Developmental Disorders, 41(11), 1556–1564. 21298331 10.1007/s10803-011-1183-3

[bibr16-1362361320932727] Bird G. Silani G. Brindley R. White S. Frith U. Singer T. (2010). Empathic brain responses in insula are modulated by levels of alexithymia but not autism. Brain, 133(5), 1515–1525. 20371509 10.1093/brain/awq060 PMC2859151

[bibr17-1362361320932727] Black M. H. Chen N. T. Lipp O. V. Bölte S. Girdler S. (2020). Complex facial emotion recognition and atypical gaze patterns in autistic adults. Autism, 24, 258–262. 31216863 10.1177/1362361319856969

[bibr18-1362361320932727] Bölte S. Poustka F. (2003). The recognition of facial affect in autistic and schizophrenic subjects and their first-degree relatives. Psychological Medicine, 33(5), 907–915. 12877405 10.1017/s0033291703007438

[bibr19-1362361320932727] Brewer R. Happé F. Cook R. Bird G. (2015). Commentary on ‘Autism, oxytocin and interoception’: Alexithymia, not Autism Spectrum Disorders, is the consequence of interoceptive failure. Neuroscience & Biobehavioral Reviews, 56, 348–353. 26192103 10.1016/j.neubiorev.2015.07.006

[bibr20-1362361320932727] Broadbent J. Galic I. Stokes M. A. (2013). Validation of autism spectrum quotient adult version in an Australian sample. Autism Research and Treatment, 2013, Article 984205. 23762552 10.1155/2013/984205 PMC3665170

[bibr21-1362361320932727] Burack J. A. Volkmar F. R. (1992). Development of low-and high-functioning autistic children. Journal of Child Psychology and Psychiatry, 33(3), 607–616. 1374418 10.1111/j.1469-7610.1992.tb00894.x

[bibr22-1362361320932727] Castelli F. (2005). Understanding emotions from standardized facial expressions in autism and normal development. Autism, 9(4), 428–449. 16155058 10.1177/1362361305056082

[bibr23-1362361320932727] Cedro A. Kokoszka A. Popiel A. Narkiewicz-Jodko W. (2001). Alexithymia in schizophrenia: An exploratory study. Psychological Reports, 89(1), 95–98. 11729558 10.2466/pr0.2001.89.1.95

[bibr24-1362361320932727] Chawarska K. Macari S. Shic F. (2013). Decreased spontaneous attention to social scenes in 6-month-old infants later diagnosed with autism spectrum disorders. Biological Psychiatry, 74(3), 195–203. 23313640 10.1016/j.biopsych.2012.11.022 PMC3646074

[bibr25-1362361320932727] Cook R. Brewer R. Shah P. Bird G. (2013). Alexithymia, not autism, predicts poor recognition of emotional facial expressions. Psychological Science, 24(5), 723–732. 23528789 10.1177/0956797612463582

[bibr26-1362361320932727] Dalton K. M. Nacewicz B. M. Johnstone T. Schaefer H. S. Gernsbacher M. A. Goldsmith H. H.. . . Davidson R. J. (2005). Gaze fixation and the neural circuitry of face processing in autism. Nature Neuroscience, 8(4), 519–526. 15750588 10.1038/nn1421 PMC4337787

[bibr27-1362361320932727] Deruelle C. Rondan C. Gepner B. Tardif C. (2004). Spatial frequency and face processing in children with autism and Asperger syndrome. Journal of Autism and Developmental Disorders, 34(2), 199–210. 15162938 10.1023/b:jadd.0000022610.09668.4c

[bibr28-1362361320932727] Durand K. Gallay M. Seigneuric A. Robichon F. Baudouin J. Y. (2007). The development of facial emotion recognition: The role of configural information. Journal of Experimental Child Psychology, 97(1), 14–27. 17291524 10.1016/j.jecp.2006.12.001

[bibr29-1362361320932727] Ekman P. Friesen W. V. (1976). Measuring facial movement. Environmental Psychology and Nonverbal Behavior, 1(1), 56–75.

[bibr30-1362361320932727] Fein D. Lueci D. Braverman M. Waterhouse L. (1992). Comprehension of affect in context in children with pervasive developmental disorders. Journal of Child Psychology and Psychiatry, 33(7), 1157–1162. 1400698 10.1111/j.1469-7610.1992.tb00935.x

[bibr31-1362361320932727] Fertuck E. A. Jekal A. Song I. Wyman B. Morris M. C. Wilson S. T.. . . Stanley B. (2009). Enhanced ‘Reading the Mind in the Eyes’ in borderline personality disorder compared to healthy controls. Psychological Medicine, 39(12), 1979–1988. 19460187 10.1017/S003329170900600X PMC3427787

[bibr32-1362361320932727] Gepner B. Deruelle C. Grynfeltt S. (2001). Motion and emotion: A novel approach to the study of face processing by young autistic children. Journal of Autism and Developmental Disorders, 31(1), 37–45. 11439752 10.1023/a:1005609629218

[bibr33-1362361320932727] Golan O. Baron-Cohen S. Hill J. (2006). The Cambridge mindreading (CAM) face-voice battery: Testing complex emotion recognition in adults with and without Asperger syndrome. Journal of Autism and Developmental Disorders, 36(2), 169–183. 16477515 10.1007/s10803-005-0057-y

[bibr34-1362361320932727] Greimel E. Schulte-Rüther M. Kamp-Becker I. Remschmidt H. Herpertz-Dahlmann B. Konrad K. (2014). Impairment in face processing in autism spectrum disorder: A developmental perspective. Journal of Neural Transmission, 121(9), 1171–1181. 24737035 10.1007/s00702-014-1206-2

[bibr35-1362361320932727] Griffin C. Lombardo M. V. Auyeung B. (2016). Alexithymia in children with and without autism spectrum disorders. Autism Research, 9(7), 773–780. 26426084 10.1002/aur.1569

[bibr36-1362361320932727] Grynberg D. Chang B. Corneille O. Maurage P. Vermeulen N. Berthoz S. Luminet O. (2012). Alexithymia and the processing of emotional facial expressions (EFEs): Systematic review, unanswered questions and further perspectives. PLOS ONE, 7(8), Article e42429. 22927931 10.1371/journal.pone.0042429 PMC3426527

[bibr37-1362361320932727] Hadjikhani N. Joseph R. M. Snyder J. Chabris C. F. Clark J. Steele S.. . . Harris G. J. (2004). Activation of the fusiform gyrus when individuals with autism spectrum disorder view faces. NeuroImage, 22(3), 1141–1150. 15219586 10.1016/j.neuroimage.2004.03.025

[bibr38-1362361320932727] Hariri A. R. Bookheimer S. Y. Mazziotta J. C. (2000). Modulating emotional responses: Effects of a neocortical network on the limbic system. NeuroReport, 11(1), 43–48. 10683827 10.1097/00001756-200001170-00009

[bibr39-1362361320932727] Harms M. B. Martin A. Wallace G. L. (2010). Facial emotion recognition in autism spectrum disorders: A review of behavioral and neuroimaging studies. Neuropsychology Review, 20(3), 290–322. 20809200 10.1007/s11065-010-9138-6

[bibr40-1362361320932727] Heaton P. Reichenbacher L. Sauter D. Allen R. Scott S. Hill E. (2012). Measuring the effects of alexithymia on perception of emotional vocalizations in autistic spectrum disorder and typical development. Psychological Medicine, 42(11), 2453–2459. 22475181 10.1017/S0033291712000621

[bibr41-1362361320932727] Heberlein A. S. Atkinson A. P. (2009). Neuroscientific evidence for simulation and shared substrates in emotion recognition: Beyond faces. Emotion Review, 1(2), 162–177.

[bibr42-1362361320932727] Hill E. Berthoz S. Frith U. (2004). Brief report: Cognitive processing of own emotions in individuals with autistic spectrum disorder and in their relatives. Journal of Autism and Developmental Disorders, 34(2), 229–235. 15162941 10.1023/b:jadd.0000022613.41399.14

[bibr43-1362361320932727] Hoekstra R. A. Bartels M. Cath D. C. Boomsma D. I. (2008). Factor structure, reliability and criterion validity of the Autism-Spectrum Quotient (AQ): A study in Dutch population and patient groups. Journal of Autism and Developmental Disorders, 38(8), 1555–1566. 18302013 10.1007/s10803-008-0538-x PMC2516538

[bibr44-1362361320932727] Hoffman E. A. Haxby J. V. (2000). Distinct representations of eye gaze and identity in the distributed human neural system for face perception. Nature Neuroscience, 3(1), Article 80. 10607399 10.1038/71152

[bibr45-1362361320932727] Homer M. Rutherford M. D. (2008). Individuals with autism can categorize facial expressions. Child Neuropsychology, 14(5), 419–437. 18720100 10.1080/09297040802291715

[bibr46-1362361320932727] Honkalampi K. Hintikka J. Tanskanen A. Lehtonen J. Viinamäki H. (2000). Depression is strongly associated with alexithymia in the general population. Journal of Psychosomatic Research, 48(1), 99–104. 10750635 10.1016/s0022-3999(99)00083-5

[bibr47-1362361320932727] Ihme K. Sacher J. Lichev V. Rosenberg N. Kugel H. Rufer M.. . . Villringer A. (2014a). Alexithymic features and the labeling of brief emotional facial expressions – An fMRI study. Neuropsychologia, 64, 289–299. 25281889 10.1016/j.neuropsychologia.2014.09.044

[bibr48-1362361320932727] Ihme K. Sacher J. Lichev V. Rosenberg N. Kugel H. Rufer M.. . . Villringer A. (2014b). Alexithymia and the labeling of facial emotions: Response slowing and increased motor and somatosensory processing. BMC Neuroscience, 15(1), 40–50. 24629094 10.1186/1471-2202-15-40 PMC4003818

[bibr49-1362361320932727] Kafetsios K. Hess U. (2019). Seeing mixed emotions: Alexithymia, emotion perception bias, and quality in dyadic interactions. Personality and Individual Differences, 137, 80–85.

[bibr50-1362361320932727] Ketelaars M. P. In’t Velt A. Mol A. Swaab H. van Rijn S. (2016). Emotion recognition and alexithymia in high functioning females with autism spectrum disorder. Research in Autism Spectrum Disorders, 21, 51–60.

[bibr51-1362361320932727] Kinnaird E. Stewart C. Tchanturia K. (2019). Investigating alexithymia in autism: A systematic review and meta-analysis. European Psychiatry, 55, 80–89. 30399531 10.1016/j.eurpsy.2018.09.004 PMC6331035

[bibr52-1362361320932727] Kothari R. Skuse D. Wakefield J. Micali N. (2013). Gender differences in the relationship between social communication and emotion recognition. Journal of the American Academy of Child & Adolescent Psychiatry, 52(11), 1148–1157. 24157389 10.1016/j.jaac.2013.08.006 PMC3989041

[bibr53-1362361320932727] Kring A. M. Elis O. (2013). Emotion deficits in people with schizophrenia. Annual Review of Clinical Psychology, 9, 409–433. 10.1146/annurev-clinpsy-050212-185538 23245340

[bibr54-1362361320932727] Lai M. C. Lombardo M. V. Ruigrok A. N. Chakrabarti B. Wheelwright S. J. Auyeung B., . . . MRC AIMS Consortium. (2012). Cognition in males and females with autism: Similarities and differences. PLOS ONE, 7(10), Article e47198. 23094036 10.1371/journal.pone.0047198 PMC3474800

[bibr55-1362361320932727] Lane R. D. Lee S. Reidel R. Weldon V. Kaszniak A. Schwartz G. E. (1996). Impaired verbal and nonverbal emotion recognition in alexithymia. Psychosomatic Medicine, 58(3), 203–210. 8771618 10.1097/00006842-199605000-00002

[bibr56-1362361320932727] Lane R. D. Sechrest L. Riedel R. Shapiro D. E. Kaszniak A. W. (2000). Pervasive emotion recognition deficit common to alexithymia and the repressive coping style. Psychosomatic Medicine, 62(4), 492–501. 10949094 10.1097/00006842-200007000-00007

[bibr57-1362361320932727] Linden W. Wen F. Paulus D. L. (1995). Measuring alexithymia: Reliability, validity, and prevalence. In Butcher J. Spielberger C. (Eds.), Advances in personality assessment (pp. 51–95). Lawrence Erlbaum.

[bibr58-1362361320932727] Loomes R. Hull L. Mandy W. P. L. (2017). What is the male-to-female ratio in autism spectrum disorder? A systematic review and meta-analysis. Journal of the American Academy of Child & Adolescent Psychiatry, 56(6), 466–474. 28545751 10.1016/j.jaac.2017.03.013

[bibr59-1362361320932727] Loth E. Garrido L. Ahmad J. Watson E. Duff A. Duchaine B. (2018). Facial expression recognition as a candidate marker for autism spectrum disorder: How frequent and severe are deficits? Molecular Autism, 9(1), Article 7. 29423133 10.1186/s13229-018-0187-7 PMC5791186

[bibr60-1362361320932727] Mériau K. Wartenburger I. Kazzer P. Prehn K. Lammers C. H. Van der Meer E.. . . Heekeren H. R. (2006). A neural network reflecting individual differences in cognitive processing of emotions during perceptual decision making. NeuroImage, 33(3), 1016–1027. 16973382 10.1016/j.neuroimage.2006.07.031

[bibr61-1362361320932727] Milosavljevic B. Leno V. C. Simonoff E. Baird G. Pickles A. Jones C. R.. . . Happé F. (2016). Alexithymia in adolescents with autism spectrum disorder: Its relationship to internalising difficulties, sensory modulation and social cognition. Journal of Autism and Developmental Disorders, 46(4), 1354–1367. 26659552 10.1007/s10803-015-2670-8

[bibr62-1362361320932727] Mul C. L. Stagg S. D. Herbelin B. Aspell J. E. (2018). The feeling of me feeling for you: Interoception, alexithymia and empathy in autism. Journal of Autism and Developmental Disorders, 48(9), 2953–2967. 29644587 10.1007/s10803-018-3564-3

[bibr63-1362361320932727] Neil L. Cappagli G. Karaminis T. Jenkins R. Pellicano E. (2016). Recognizing the same face in different contexts: Testing within-person face recognition in typical development and in autism. Journal of Experimental Child Psychology, 143, 139–153. 26615971 10.1016/j.jecp.2015.09.029 PMC4722798

[bibr64-1362361320932727] Nemiah J. Freyberger H. Sifneos P. E. (1976). Alexithymia: A view of the psychosomatic process. In Hill O.W. (Ed.), Modern tends in psychosomatic medicine (Vol. 3, pp. 430–439). Butterworths.

[bibr65-1362361320932727] Oakley B. F. Brewer R. Bird G. Catmur C. (2016). Theory of mind is not theory of emotion: A cautionary note on the Reading the Mind in the Eyes Test. Journal of Abnormal Psychology, 125(6), 818–823. 27505409 10.1037/abn0000182 PMC4976760

[bibr66-1362361320932727] O’Connor K. Hamm J. P. Kirk I. J. (2005). The neurophysiological correlates of face processing in adults and children with Asperger’s syndrome. Brain and Cognition, 59(1), 82–95. 16009478 10.1016/j.bandc.2005.05.004

[bibr67-1362361320932727] Ozonoff S. Pennington B. F. Rogers S. J. (1990). Are there emotion perception deficits in young autistic children? Journal of Child Psychology and Psychiatry, 31(3), 343–361. 2318918 10.1111/j.1469-7610.1990.tb01574.x

[bibr68-1362361320932727] Parker J. D. Taylor G. J. Bagby M. (1993). Alexithymia and the recognition of facial expressions of emotion. Psychotherapy and Psychosomatics, 59(3–4), 197–202. 8416096 10.1159/000288664

[bibr69-1362361320932727] Parker P. D. Prkachin K. M. Prkachin G. C. (2005). Processing of facial expressions of negative emotion in alexithymia: The influence of temporal constraint. Journal of Personality, 73(4), 1087–1107. 15958145 10.1111/j.1467-6494.2005.00339.x

[bibr70-1362361320932727] Peñuelas-Calvo I. Sareen A. Sevilla-Llewellyn-Jones J. Fernández-Berrocal P. (2019). The ‘Reading the Mind in the Eyes’ Test in autism-spectrum disorders comparison with healthy controls: A systematic review and meta-analysis. Journal of Autism and Developmental Disorders, 49(3), 1048–1061. 30406435 10.1007/s10803-018-3814-4

[bibr71-1362361320932727] Peterson C. Slaughter V. Moore C. Wellman H. M. (2016). Peer social skills and theory of mind in children with autism, deafness, or typical development. Developmental Psychology, 52(1), 46–57. 26524383 10.1037/a0039833

[bibr72-1362361320932727] Poquérusse J. Pastore L. Dellantonio S. Esposito G. (2018). Alexithymia and autism spectrum disorder: A complex relationship. Frontiers in Psychology, 9, Article 1196. 30065681 10.3389/fpsyg.2018.01196 PMC6056680

[bibr73-1362361320932727] Preston S. D. De Waal F. B. (2002). Empathy: Its ultimate and proximate bases. Behavioral and Brain Sciences, 25(1), 1–20. 12625087 10.1017/s0140525x02000018

[bibr74-1362361320932727] Prkachin G. C. Casey C. Prkachin K. M. (2009). Alexithymia and perception of facial expressions of emotion. Personality and Individual Differences, 46(4), 412–417.

[bibr75-1362361320932727] Qualtrics. (2019). Qualtrics software. https://www.qualtrics.com

[bibr76-1362361320932727] Riby D. M. Doherty-Sneddon G. Bruce V. (2009). The eyes or the mouth? Feature salience and unfamiliar face processing in Williams syndrome and autism. The Quarterly Journal of Experimental Psychology, 62(1), 189–203. 18609381 10.1080/17470210701855629

[bibr77-1362361320932727] Rivet T. T. Matson J. L. (2011). Review of gender differences in core symptomatology in autism spectrum disorders. Research in Autism Spectrum Disorders, 5(3), 957–976.

[bibr78-1362361320932727] Schlegel K. Scherer K. R. (2016). Introducing a short version of the Geneva Emotion Recognition Test (GERT-S): Psychometric properties and construct validation. Behavior Research Methods, 48(4), 1383–1392. 26416137 10.3758/s13428-015-0646-4

[bibr79-1362361320932727] Schultz R. T. (2005). Developmental deficits in social perception in autism: The role of the amygdala and fusiform face area. International Journal of Developmental Neuroscience, 23(2–3), 125–141. 15749240 10.1016/j.ijdevneu.2004.12.012

[bibr80-1362361320932727] Schultz R. T. Grelotti D. J. Klin A. Kleinman J. Van der Gaag C. Marois R. Skudlarski P. (2003). The role of the fusiform face area in social cognition: Implications for the pathobiology of autism. Philosophical Transactions of the Royal Society B: Biological Sciences, 358(1430), 415–427. 10.1098/rstb.2002.1208 PMC1693125 12639338

[bibr81-1362361320932727] Shah P. Hall R. Catmur C. Bird G. (2016). Alexithymia, not autism, is associated with impaired interoception. Cortex, 81, 215–220. 27253723 10.1016/j.cortex.2016.03.021 PMC4962768

[bibr82-1362361320932727] Shanok N. A. Jones N. A. Lucas N. N. (2019). The nature of facial emotion recognition impairments in children on the autism spectrum. Child Psychiatry & Human Development, 2019, 661–667. 10.1007/s10578-019-00870-z 30756220

[bibr83-1362361320932727] Stephenson K. G. Luke S. G. South M. (2019). Separate contributions of autistic traits and anxious apprehension, but not alexithymia, to emotion processing in faces. Autism, 23(7), 1830–1842. 30848668 10.1177/1362361319830090

[bibr84-1362361320932727] Sucksmith E. Allison C. Baron-Cohen S. Chakrabarti B. Hoekstra R. A. (2013). Empathy and emotion recognition in people with autism, first-degree relatives, and controls. Neuropsychologia, 51(1), 98–105. 23174401 10.1016/j.neuropsychologia.2012.11.013 PMC6345368

[bibr85-1362361320932727] Swart M. Kortekaas R. Aleman A. (2009). Dealing with feelings: Characterization of trait alexithymia on emotion regulation strategies and cognitive-emotional processing. PLOS ONE, 4(6), Article e5751. 19492045 10.1371/journal.pone.0005751 PMC2685011

[bibr86-1362361320932727] Tanaka J. W. Wolf J. M. Klaiman C. Koenig K. Cockburn J. Herlihy L.. . . Kaiser M. D. (2012). The perception and identification of facial emotions in individuals with autism spectrum disorders using the Let’s Face It! Emotion Skills Battery. Journal of Child Psychology and Psychiatry, 53(12), 1259–1267. 22780332 10.1111/j.1469-7610.2012.02571.x PMC3505257

[bibr87-1362361320932727] Taruffi L. Allen R. Downing J. Heaton P. (2017). Individual differences in music-perceived emotions: The influence of externally oriented thinking. Music Perception: An Interdisciplinary Journal, 34(3), 253–266.

[bibr88-1362361320932727] Teunisse J. P. de Gelder B. (2001). Impaired categorical perception of facial expressions in high-functioning adolescents with autism. Child Neuropsychology, 7(1), 1–14. 11815876 10.1076/chin.7.1.1.3150

[bibr89-1362361320932727] Tracy J. L. Robins R. W. Schriber R. A. Solomon M. (2011). Is emotion recognition impaired in individuals with autism spectrum disorders? Journal of Autism and Developmental Disorders, 41(1), 102–109. 20464465 10.1007/s10803-010-1030-y PMC3005106

[bibr90-1362361320932727] Travers B. G. Bigler E. D. Tromp D. P. Adluru N. Froehlich A. L. Ennis C.. . . Lainhart J. E. (2014). Longitudinal processing speed impairments in males with autism and the effects of white matter microstructure. Neuropsychologia, 53, 137–145. 24269298 10.1016/j.neuropsychologia.2013.11.008 PMC3946881

[bibr91-1362361320932727] Uljarevic M. Hamilton A. (2013). Recognition of emotions in autism: A formal meta-analysis. Journal of Autism and Developmental Disorders, 43(7), 1517–1526. 23114566 10.1007/s10803-012-1695-5

[bibr92-1362361320932727] Vermeulen N. Luminet O. De Sousa M. C. Campanella S. (2008). Categorical perception of anger is disrupted in alexithymia: Evidence from a visual ERP study. Cognition and Emotion, 22(6), 1052–1067.

[bibr93-1362361320932727] Weigelt S. Koldewyn K. Kanwisher N. (2012). Face identity recognition in autism spectrum disorders: A review of behavioral studies. Neuroscience & Biobehavioral Reviews, 36(3), 1060–1084. 22212588 10.1016/j.neubiorev.2011.12.008

[bibr94-1362361320932727] Wingbermühle E. Theunissen H. Verhoeven W. M. Kessels R. P. Egger J. I. (2012). The neurocognition of alexithymia: Evidence from neuropsychological and neuroimaging studies. Acta Neuropsychiatrica, 24(2), 67–80. 26952949 10.1111/j.1601-5215.2011.00613.x

[bibr95-1362361320932727] Wolf J. M. Tanaka J. W. Klaiman C. Cockburn J. Herlihy L. Brown C.. . . Schultz R. T. (2008). Specific impairment of face-processing abilities in children with autism spectrum disorder using the Let’s Face It! skills battery. Autism Research, 1(6), 329–340. 19360688 10.1002/aur.56 PMC4589218

[bibr96-1362361320932727] Zonnevijlle-Bendek M. J. S. Van Goozen S. H. M. Cohen-Kettenis P. T. Van Elburg A. Van Engeland H. (2002). Do adolescent anorexia nervosa patients have deficits in emotional functioning? European Child & Adolescent Psychiatry, 11(1), 38–42. 11942427 10.1007/s007870200006

